# Distribution and pattern of hand fractures in children and adolescents

**DOI:** 10.1007/s00431-023-04915-3

**Published:** 2023-04-05

**Authors:** Christoph Arneitz, Claudia Bartik, Claus-Uwe Weitzer, Barbara Schmidt, Paolo Gasparella, Sebastian Tschauner, Christoph Castellani, Holger Till, Georg Singer

**Affiliations:** 1 Department of Paediatric and Adolescent Surgery, Medical University of Graz, Auenbruggerplatz 34, 8036 Graz, Austria; 2 Division of Paediatric Radiology, Department of Radiology, Medical University of Graz, Graz, Austria

**Keywords:** Hand fractures, Children, Adolescents

## Abstract

Hand fractures represent commonly encountered injuries in pediatric patients. However, due to modern means of mobility and product safety, the occurrence and distribution of these fractures have changed during the last decades. Therefore, it was the aim of this study to present an update of the epidemiology, pattern, and treatment of hand fractures in a large pediatric cohort. All patients aged between 0 and 17 years treated in our Department in 2019 with fractures of the phalanges, metacarpus, or carpus were included. The medical records were reviewed for age, gender, injury mechanism, fracture localization, season, and treatment. Patients were divided into three different age groups (0–5, 6–12, and 13–17 years). A total of 731 patients with 761 hand fractures were treated during the 1-year study period. The mean age was 11.1 ± 3.5 years, and the majority was male (65%). Male patients were significantly older compared to female patients (*p* = 0.008). Also, 78.7% of the fractures affected the phalanges, 17.6% the metacarpals, and 3.7% the carpal bones. The proximal phalanges were the most commonly fractured bones (41.5%). Patients with fractures of the carpus were significantly older compared to children sustaining fractures of the metacarpus or phalangeal bones (*p* < 0.001). Sixteen percent of our patients were treated surgically; these patients were significantly older compared to conservatively treated patients (*p* = 0.011).

*  Conclusion*: The epidemiology, mechanisms of injury, distribution, and treatment of hand fractures significantly varies among different age groups. This knowledge is of importance for educational purposes of younger colleagues entrusted with care of children and adolescents as well as development of effective prevention strategies.

**Table:** 

**What is Known:** *• Pediatric hand fractures represent the second most common fractures in children.* *• The epidemiology of pediatric hand fractures has changed during the last decades and therefore there is a need for an update regarding distribution and epidemiology of pediatric hand fractures.*	
**What is New:** *• In this retrospective cohort study, 761 pediatric hand fractures of 731 patients were analyzed in detail.* *• The main mechanisms of younger patients were entrapment injuries, older children most commonly sustained their fractures due to ball sport injuries. There was an increasing rate of metacarpal and carpal fractures with increasing age, and these fractures had to be treated operatively more often than phalangeal fractures.*

**Supplementary Information:**

The online version contains supplementary material available at 10.1007/s00431-023-04915-3.

## Introduction

Pediatric hand fractures represent the second most common fractures in children with an overall annual incidence of 26.4 up to 44.8 per 10,000 children [1, 2]. Hand fractures usually peak in two age groups, toddlers and adolescents. In toddlers, injuries of the hand are frequently secondary to a crush injury or entrapment. The second peak of incidence occurs between 13 and 15 years of age and is related to increased participation in sports activities [1, 3, 4]. However, there is an ongoing discussion about the most commonly fractured bone of the child’s hand [4]. The proximal phalanx seems to be the most frequently affected [5, 6], but in younger children, distal tuft fractures can be even more common [7, 8]. Moreover, pediatric hand fractures show a clear male predominance, except in toddlers [8] and are a domain of conservative fracture treatment [1, 9].

The epidemiology of pediatric hand fractures has changed during the last decades due to modern means of mobility and product safety, and therefore there is a need for an update regarding distribution and epidemiology of pediatric hand fractures in order to adapt effective prevention strategies [2, 10]. Additionally, current knowledge of the distribution, pattern, and treatment of pediatric hand fractures are key for educational purposes of younger colleagues entrusted with care of children and adolescents as well as allocation of healthcare resources [2].

Therefore, it was the aim of this study to perform an in-depth analysis assessing the fracture epidemiology, mechanisms of injury, distribution, and treatment of hand fractures in a large cohort of pediatric patients. This study intended to highlight the importance of meticulous assessment of pediatric finger and hand fractures when presented to primary care, thereby initiating an individual approach for every single injury considering up-to-date mechanisms of trauma.

## Patients and methods

The Department of Pediatric Surgery in Graz represents the only Level I Trauma Referral Center for children in the province of Styria, Austria, covering a population of 1.2 million. Following approval of the local Ethics Committee (EK 32–302 ex 19/20), we conducted a retrospective study of all patients between 0 and 17 years treated in our department in 2019 with fractures of the phalanges, metacarpus, or carpus. The medical records of these patients were reviewed for age, gender, season, injury mechanism, fracture localization, and treatment. Patients were divided into 3 different age groups (0–5, 6–12, and 13–17 years). Injury mechanisms were grouped as falls, ball sports, other sports activities, vehicle associated injures (including skateboards, bicycles, and mopeds), leisure time activities (such as trampoline and playground associated injuries), entrapment, violence related injuries (scuffle, kicks, collision with other children), or unknown/other causes. Indications for operative treatment were open fractures, intraarticular fractures with displacement or affection of more than 20% of the articular surface, and instable fractures with significant, visible rotational deformity (> 10°) or significant angulation.

### Statistical analysis

All data were entered into an Excel 2019® (Microsoft Corporation, Microsoft Excel [Internet]. 2018, USA) spreadsheet and then transferred to SPSS Statistics 21^©^ (IBM Corp. Released 2012. IBM SPSS Statistics for Windows, Version 21.0. Armonk, NY: IBM Corp) for statistical analysis. Metric data are displayed as mean, range, and/or standard deviation. Statistical group comparison of metric data was performed with the Mann–Whitney *U* test. Age comparison of different fracture localizations was performed with the Kruskal–Wallis test and post hoc tests applying a Bonferroni correction. Categorical data were compared with the Chi-squared test. Explorative statistical significance was defined as *p* < 0.05.

## Results

**Fig. 1:**
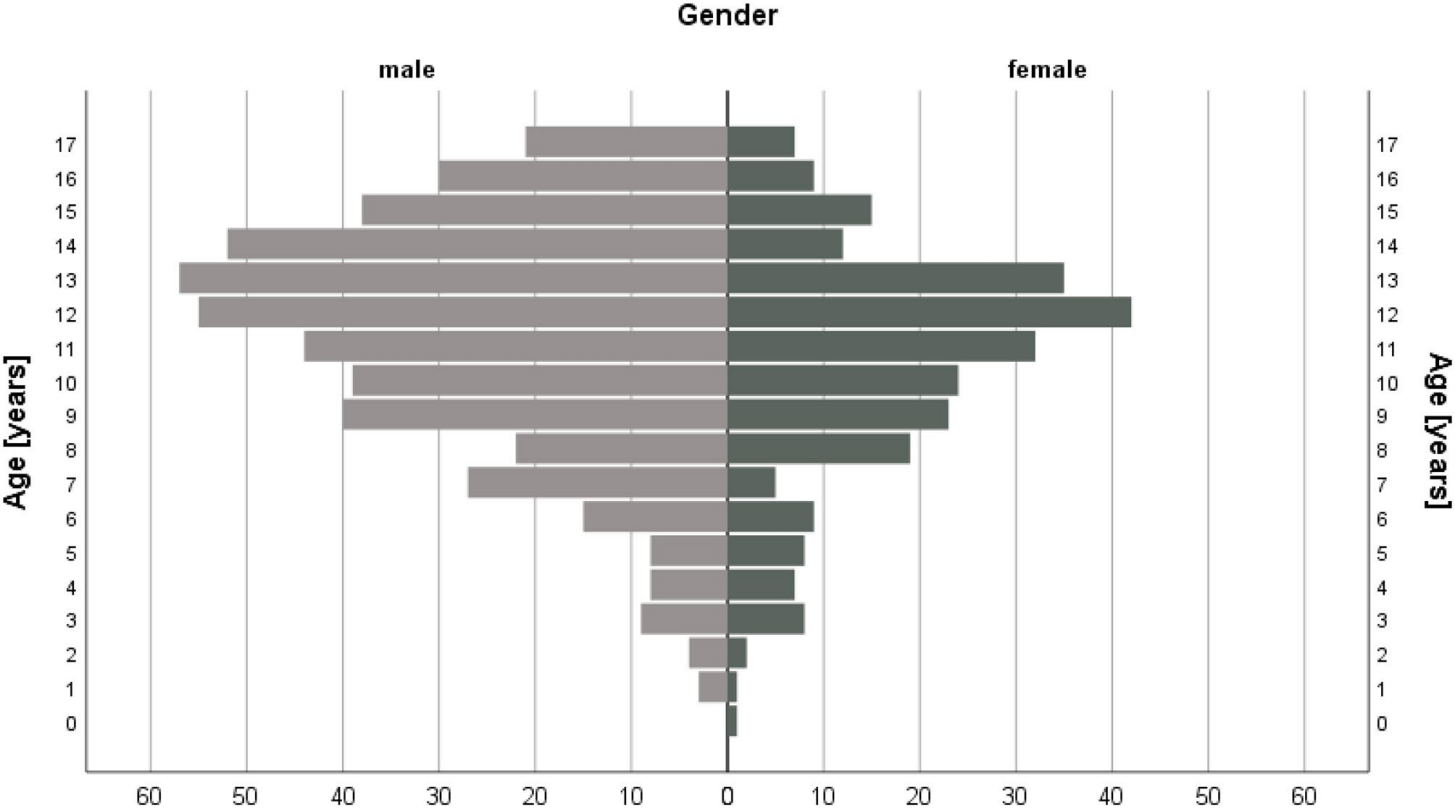
Gender distribution of 731 pediatric patients with hand fractures according to age

During the study period of 12 months, 731 patients (65% male, 35% female) with a mean age of 11.1 years (range 0–17, SD 3.5 years) were treated with hand fractures. The gender distribution according to age is shown in Fig. 1. Male patients were significantly older compared to female patients (male mean age 11.3 ± 3.5 years vs. female mean age 10.6 ± 3.5 years, *p* = 0.008).

The main mechanisms of injury were ball sports (34.9%), followed by entrapment (15.2%), and falls (15%). The injury mechanisms are listed in Table 1. Pediatric hand fractures occurred relatively regularly during the year with peaks in spring and autumn (spring *n* = 209, 28.6%; summer *n* = 148, 20.2%; autumn *n* = 218, 29.8%; winter *n* = 156, 21.4%).

**Table 1:** Main injury mechanisms of 731 pediatric patients sustaining hand fractures

	***n***	**%**
Ball sports	255	34.9
Entrapment	111	15.2
Falls	110	15
Leisure time activities	65	8.9
Other sports activities	63	8.6
Vehicle associated	47	6.4
Violence related	33	4.5
Others/unknown	47	6.5
**Total**	**731**	**100**

The vast majority of the patients sustained one fracture (*n* = 703, 96.2%). Twenty-eight children presented with more than one fracture (*n* = 26 two fractures, *n* = 2 three fractures) resulting in a total number of fractures of *n* = 761. Also, 55.1% (*n* = 419) occurred on the right hand and 44.9% (*n* = 342) on the left side.

**Fig. 2:**
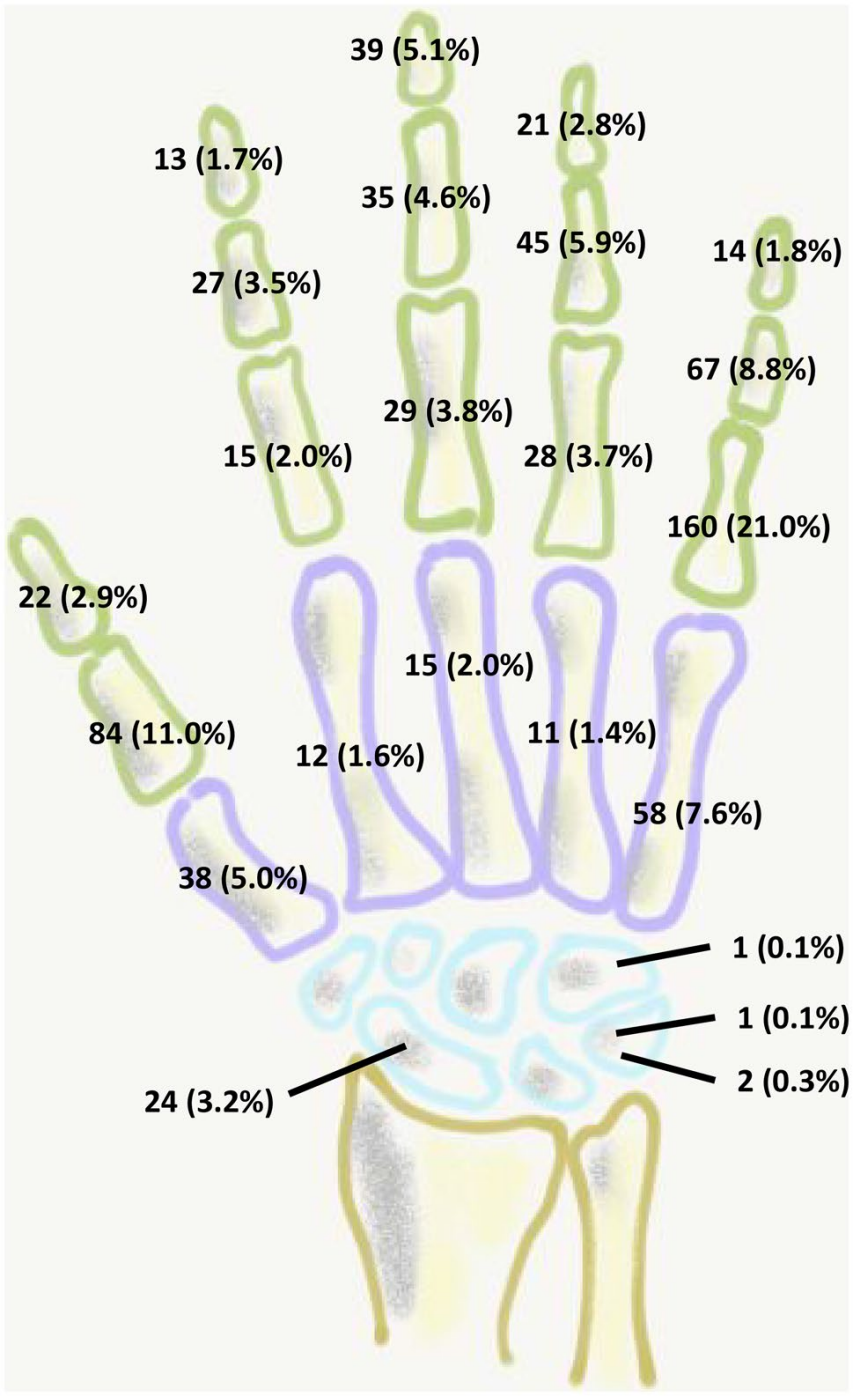
Distribution of 761 pediatric hand fractures

The vast majority of the fractures affected phalangeal bones (*n* = 599, 78.7%). Fractures of metacarpals and carpal bones were diagnosed 134 (17.6%) and 28 (3.7%) times, respectively. The most commonly fractured bone was the proximal phalanx of the fifth finger (*n* = 160, 21%), followed by the proximal phalanx of the thumb (*n* = 84, 11%) and the middle phalanx of the fifth ray (*n* = 67, 8.8%). The most frequently injured rays were the fifth (*n* = 299, 39.2%) and first (*n* = 144, 18.9%).The exact distribution of the fractured bones is depicted in Fig. 2.

### Age group analysis

For further analysis, patients were divided into 3 different age groups. Fifty-nine patients (8.1%) were aged 0–5 years, 396 (54.2%) were aged between 6 and 12 years, and 276 (37.7%) were adolescents [13-17years].

The proportion of male patients constantly rose with increasing age starting from 54.2% in children aged 0–5 years to 61.1% in 6–12-year olds and 71.7% in adolescents. These differences were statistically significant (*p* = 0.004).

While the main mechanism of injury was entrapment (49.2%) in children aged between 0 and 5 years, patients aged from 6 to 12 years and 13 to 17 years most often sustained the fracture during ball sports (34.8% and 41.5%, respectively). A detailed presentation of the mechanisms of injury is shown in Table 2.

**Table 2:** Main injury mechanisms according to three age groups (0–5 years *n* = 59, 6–12 years *n* = 396, 13–17 years *n* = 276); the two most common mechanisms are marked in bold

	**0–5 years**		**6–12 years**		**13–17 years**	
	***n***	**%**	***n***	**%**	***n***	**%**
Ball sports	3	5.1	**138**	**34.8**	**114**	**41.5**
Falls	**14**	**23.7**	**62**	**15.7**	**34**	**12.3**
Entrapment	**29**	**49.2**	53	13.4	29	10.5
Leisure time activities	5	8.5	48	12.1	12	4.3
Other sports activities	1	1.7	28	7.1	34	12.3
Vehicle associated	2	3.4	16	4.0	29	10.5
Violence related	1	1.7	20	5.1	12	4.3
Others/unknown	4	6.7	31	7.8	12	4.3
**Total**	**59**	**100**	**396**	**100**	**276**	**100**

91.5% (*n* = 54) of 0–5 year-olds sustained one fracture and 8.5% (*n* = 5) two fractures. Children aged between 6 and 12 years presented with single fractures in 97.5% (*n* = 386), two hand fractures in 2.3% (*n* = 9), and three fractures in 0.2% (*n* = 1). Adolescents [13-17years] sustained one fracture in 95.3% (*n* = 263), two in 4.3% (*n* = 12), and three fractures in 0.4% (*n* = 1).

**Fig. 3:**
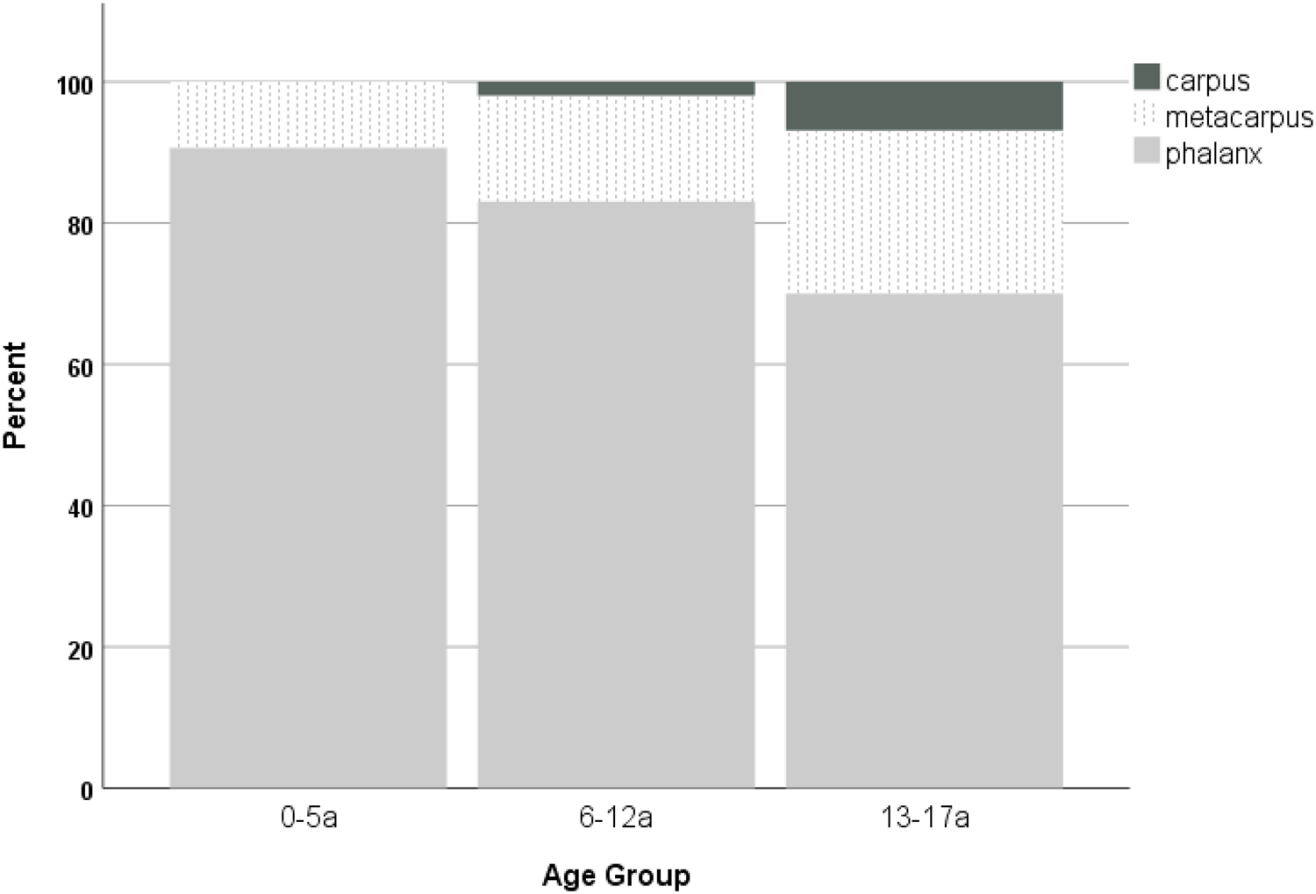
Distribution of fracture location according to age group

There was a significantly different distribution between the affected region according to age group (*p* < 0.001). While no carpal fractures occurred in children aged 0–5 years, these fractures were diagnosed in 6.9% of adolescents. In contrary, phalangeal fractures were seen in 90.6% of 0–5-year olds and declined to a rate of 70% in adolescents. The proportion of metacarpal fractures increased with increasing age. The detailed proportions are depicted in Fig. 3.

The most commonly affected bones in children aged 0–5 years was the proximal fifth phalanx (14.1%, *n* = 9), the proximal phalanx of the thumb (14.1%, *n* = 9), and the distal phalanx of the thumb (12.5%, *n* = 8). Likewise, in children 6–12 years old, almost one-third presented with fractures of the proximal phalanx of the fifth finger (30%, *n* = 122). The most commonly encountered fractures in adolescents affected the proximal phalanx of the thumb (10.7%, *n* = 31), the fifth metacarpal (10.3%, *n* = 30), and the proximal phalanx of the fifth finger (10%, *n* = 29). A detailed distribution of the fractured bones in the three different age groups is presented in Supplementary Table 1.

**Fig. 4:**
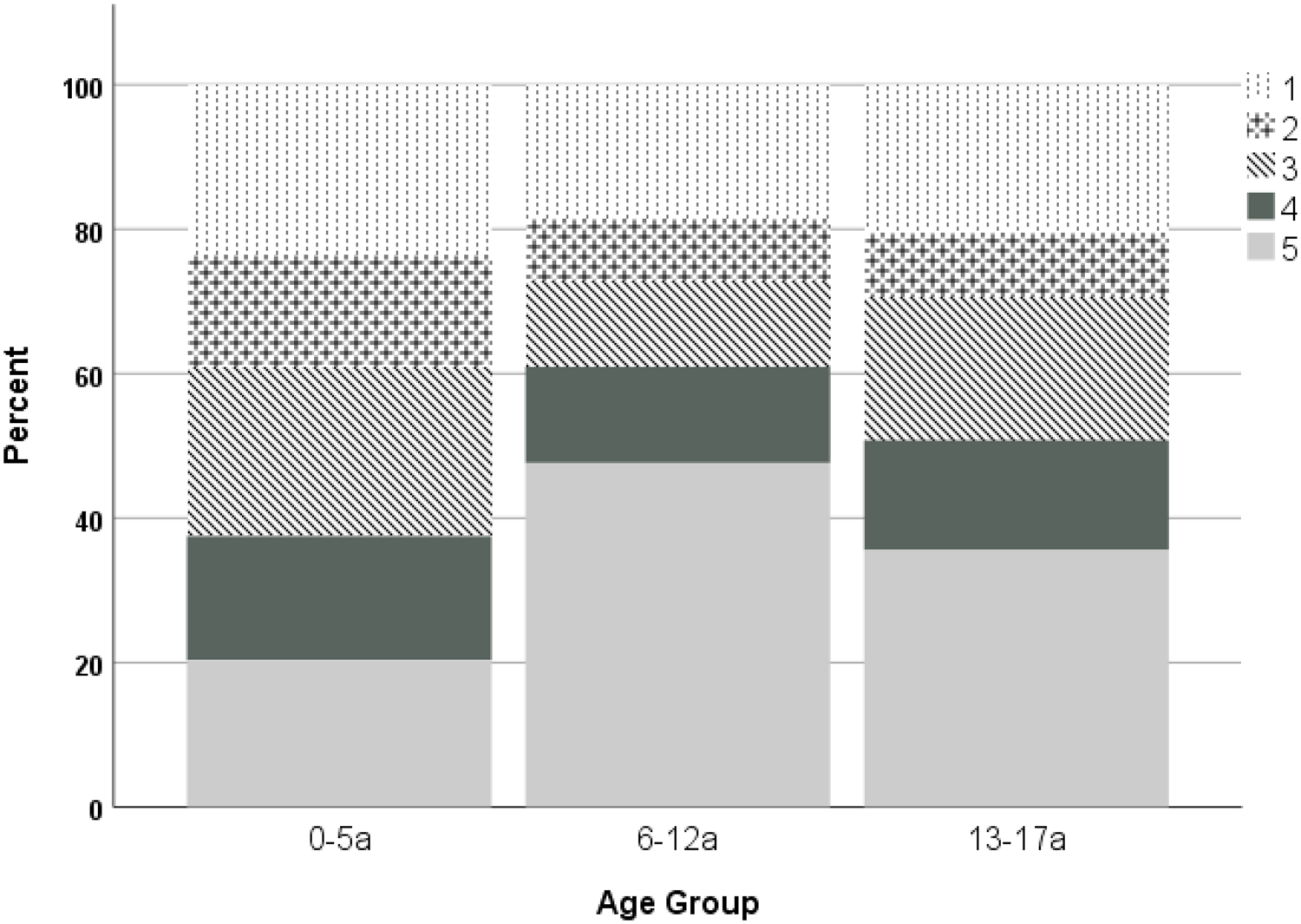
Affected rays in the different ag groups studied

There was a significantly different distribution of the affected rays according to the age groups (*p* = 0.001). While the thumb and the third ray were most often affected in children aged 0–5 years (each 24.4%, *n* = 15), the fifth ray was the predominant ray in the other two age groups (6–12 years, 47.6%, *n* = 190; 13–17 years, 35.6%, *n* = 96). The detailed results are depicted in Fig. 4.

### Anatomic fracture location analysis

Fractures most often affected phalangeal bones (*n* = 599, 78.7%) followed by metacarpals in 134 (17.6%) and carpal bones in 28 (3.7%) cases.

Patients with fractures of the carpus were significantly older (mean age 13.9 ± 2.0 years) compared to children sustaining fractures of the metacarpus (mean age 12.1 ± 3.4 years, *p* = 0.009) or phalangeal bones (mean age 10.7 ± 3.6 years, *p* < 0.001).

There was significantly different gender distribution concerning the affected anatomic fracture location (*p* < 0.001). While both carpal and metacarpal fractures were typical male injuries (82.1% and 84.3% male, respectively), the male predominance was lower for fractures of the phalanges (60.1%).

In almost half of the cases (46.4%), carpal fractures were fall-related. The main mechanisms causing metacarpal fractures were falls and entrapment (27.6% and 17.9%). Phalangeal fractures were predominantly ball sport related (39.6%). Detailed results are shown in Table 3.

**Table 3:** Main injury mechanisms according to the three anatomic locations; the two most common mechanisms are marked in bold

	**Carpus**		**Metacarpus**		**Phalanges**	
	***n***	**%**	***n***	**%**	***n***	**%**
Ball sports	2	7.1	21	15.7	**237**	**39.6**
Falls	**13**	**46.4**	**37**	**27.6**	65	10.9
Entrapment	0	0	**24**	**17.9**	**97**	**16.2**
Leisure time activities	0	0	11	8.2	56	9.3
Other sports activities	3	10.7	11	8.2	51	8.5
Vehicle associated	**10**	**35.7**	15	11.2	26	4.3
Violence related	0	0	7	5.2	26	4.3
Others/unknown	0	0	8	6.0	41	6.9
**Total**	**28**	**100**	**134**	**100**	**599**	**100**

### Operative treatment

A total of 122 fractures (16%) in 117 patients (16%) had to be treated surgically; these patients were significantly older compared to conservatively treated patients (surgical group mean age 11.7 ± 3.9 years vs. conservative group mean age 10.9 ± 3.5 years, *p* = 0.011).

The necessity of operative treatment was significantly different between the age groups (*p* = 0.019). While 13 fractures in children aged 0–5 years (20.3%) were treated operatively, the percentage of fractures necessitating operative treatment was 12.5% (*n* = 51) in 6–12 years old and 20% (*n* = 58) in teenager (13–17 years).

The rate of operative treatment was 7.1% (*n* = 2) for carpal fractures, 20.9% (*n* = 28) for metacarpal, and 15.4% (*n* = 92) for phalangeal fractures.

The main indications for surgical treatment were displacement (*n* = 89, 73%), open fractures (*n* = 10, 8.2%), malrotation (n = 6, 4.9%), and intraarticular fractures (*n* = 6, 4.9%). Other causes occurred rarely (Supplementary Table 2).

Almost two-thirds of the fractures were treated with closed reduction and immobilization (*n* = 81, 66.4%). A detailed description of the surgical management is presented in Table 4.

**Table 4:** Surgical treatment of 122 pediatric hand fractures

	***n***	**%**
Closed reduction and immobilization	81	66.5
Closed reduction and pin	10	8.2
Closed reduction and intramedullary nail	2	1.6
Open reduction	1	0.8
Open reduction and pin	8	6.6
Open reduction and screw	6	4.9
Open reduction and plate	1	0.8
Open reduction and external fixation	2	1.6
Arthrodesis	1	0.8
Wound closure	10	8.2
**Total**	**122**	**100**

In 16 cases (19.8%), a secondary displacement or malrotation was seen following closed reduction and immobilization. Thirteen of them had another closed reduction; 3 required a surgical intervention with open reduction and pin (*n* = 1), de-rotation osteotomy and plate osteosynthesis (*n* = 1), and open reduction (*n* = 1).

## Discussion

In this retrospective cohort study, 761 pediatric hand fractures of 731 patients were analyzed in detail. Male patients were significantly older than females. Moreover, while the main mechanisms of younger patients were entrapment injuries, older children most commonly sustained their fractures due to ball sport injuries. There was also an increasing rate of metacarpal and carpal fractures with increasing age, and these fractures had to be treated operatively more often than phalangeal fractures.

In a large review of the frequency of pediatric hand fractures, the mean age of the patients was 12.2 years with most of the fractures occurring in patients aged between 12 and 17 years [5]. These results are comparable to our findings with a mean age of 11.1 years and an incidence peak of patients aged 12 and 13 years. In an interesting epidemiological study comparing time trends of pediatric hand fractures from 1950 to 1955 and 2005 to 2006 performed in Malmö, Sweden, by Lempesis and coworkers, the authors found a peak incidence at an age of 12–13 years in the later years as compared to 14–15 years in the earlier time period [2]. These changes underline the necessity of up-to-date data regarding pediatric hand fractures. Thus, modern healthcare systems should primarily focus on frequent medical problems such as hand fractures by increased education and adapt treatment strategies to modern epidemiological requirements.

Pediatric hand fractures show a clear male preponderance and are three times more common in boys [11]. Exempted from this are younger children, in whom hand fractures occur almost equally distributed in boys and girls (*n* = 32 vs. *n* = 27, compare Fig. 1). This could also be shown in a previous study, where even 66% of the patients below 5 years of age were female [8]. The increasing rate of males in patients from school age upwards (reaching more than two thirds in adolescents in our study) may be explained by the interest in differing sports activities and a more risk taking behavior of boys [7].

Pediatric hand fractures were regularly distributed during a 1-year period without significant differences between the different seasons, which is according to the recent literature [12, 13]. No significant differences in the month of occurrence were observed for hand fractures based on location or allocated for different age groups in a retrospective cross sectional study of 1441 post-traumatic hand radiopgraphs [12]. This was also seen in a prospective survey of 161 hand fractures with a mean patients’ age of 24 (range 2–76) [13].

Injuries sustained at ball sports, followed by falls or entrapment, were the most common mechanism leading to hand fractures. These findings are consistent with a variety of other reports describing sporting injuries as the most common cause of hand fractures [1, 14, 15]. Analysis of the different age groups revealed that entrapment was the most frequent mechanism of injury in children aged between 0 and 5 years. This finding corresponds to other reports describing distal tuft fractures as the most common fractures in the 0 to 4-year age group [8] and can be explained by the natural curiosity combined with the still developing fine motors function of these children. In contrast to other reports, punch related injuries were not as common in our study population (17% of all fractures in Young et al. [1] were punch related vs. 4.5% in our patient population).

The most commonly fractured bone was the proximal phalanx of the fifth finger and the thumb accounting for almost a third of the fractures. Moreover, the fifth (39.3%) and first (18.9%) rays were most frequently injured suggesting that the border digits are more prone to injuries compared to the central rays. These results correspond well to previously published studies [3, 5, 6, 8]. One of the largest cohorts was reported by Kreutz-Rodrigues and coworkers in 2020 including 4356 pediatric hand fractures; the authors found that almost half of the fractures occurred in the proximal/middle phalanx, but did neither separate these two bones nor report the affected ray in their statistical analysis [5]. Since this study reflects the epidemiological characteristics of the USA, the data is not consistent with the European findings in our investigation. For example, the numbers of closed and open reductions indicate that simple immobilization with splinting of cast application is less frequent compared to our results. Another prospective observational study over a 3-month period, including 70 consecutive children with 83 hand fractures, was reported by Lui et al. [6]. The authors also found the proximal phalanx as the most frequently affected bone, but also stated that metacarpal fractures were most common in 1- to 3-year-old children (47%) [6]. However, their case numbers were small (*n* = 8). In contrast to these findings, we rarely found fractures of the metacarpus in children below 6 years of age (9.4%). In their analysis of 293 fractures in 280 children, Rajesh and colleagues have found distal tuft fractures as the most frequently occurring fractures and have reported only one fracture of the proximal phalanx in 0 to 4-year old patients [8]. This is in contrast to our results, including 23 fractures of the proximal phalanx in children below 6 years of age. However, the number of distal fractures (*n* = 27) was almost equivalent, confirming that fractures of the distal phalanx are more often diagnosed in younger patients [16].

We also found a statistically significant difference of the mean ages of children sustaining fractures of the phalanges, the metacarpus and carpus, confirming previously published results that with increasing age of the patients the rate of proximal fractures increases [3, 8]. While patients with fractures of the carpus were significantly older, no carpal fracture was found in the age group 0–5 years. Carpal fractures mainly affected the scaphoid (*n* = 24 out of 28, 85.7%), whereas fractures of the triquetrum, pisiforme, and hamatum were rarities.

According to the literature, pediatric hand fractures rarely require surgical management [1, 5, 9, 16]. Surgically treated patients were significantly older with a mean age of 11.7 years, and the indication for surgical treatment was independent of the affected region. The rate of surgical interventions in pediatric hand fractures varied between 10 and 20% in a literature review and was usually low due to great remodeling potential of children [6]. The rate of surgical fixation was even lower in a retrospective review of 283 children with 303 hand fractures, accounting for only 5% and was associated with a higher risk of complications [1]. A recently published study reported a surgical rate of pediatric hand fractures below 10%; open fractures, fracture rotation, fractures of the distal phalanx, multiple fractures, oblique pattern, comminution, displacement of more than 2 mm, intra-articular fractures, and angulation of more than 15° were found to be significant risk factors for surgical intervention [9]. The rate of fractures necessitating some kind of intervention was 16%. Most of these fractures, however, were treated with closed reduction and immobilization supporting the findings of previous studies that the majority of pediatric hand fractures yield excellent results following conservative and operative treatment [17]. However, there is a certain subset of fractures including fractures with extensive soft tissue involvement, complex intraarticular fractures, and phalangeal neck fractures that is associated with an increased appearance of long-term sequelae [18]. Therefore, healthcare personnel entrusted with pediatric hand fractures should be aware of these problematic fractures. Moreover, there are reports showing that clinically significant fracture displacement is rare after operative reduction and fixation in the pediatric age group and therefore authors suggest to refrain from routine postoperative radiographs [19]. Our data, however, reveal that following reduction without stabilization leads to a secondary displacement in 19.8% of the cases suggesting that routine follow-up X-rays seems to be necessary in this group of patients.

Objective parameters and indication criteria for fractures that require surgical management are still lacking [16]. Pediatric hand fractures can be treated operatively with closed reduction with percutaneous pinning and usually show a complete fracture healing within 3 to 4 weeks, with the scaphoid as a notable exception [20].

Limitations of the present study include the retrospective study design and the possibility of incomplete data due to missing records or ICD codes. Nevertheless, we are able to present an in-depth analysis of a large study cohort of pediatric patients with hand fractures treated in a 1-year period.

In conclusion, the epidemiology, mechanisms of injury, distribution, and treatment of hand fractures significantly varies among different age groups. The management of pediatric hand fractures requires correct diagnosis and knowledge of their common regions. This knowledge is of importance for educational purposes of younger colleagues entrusted with care of children and adolescents as well as allocation of healthcare resources.

## Supplementary Information

Below is the link to the electronic supplementary material.

Supplementary file1 (DOCX 19 KB)

Supplementary file2 (DOCX 14 KB)

## Data Availability

The data that support the findings of this study are available from the corresponding author, GS, upon reasonable request.
