# Supplementary material for: Distribution and pattern of hand fractures in children and adolescents
Source: Eur J Pediatr. 2023 Apr 5;182(6):2785–92. doi: 10.1007/s00431-023-04915-3 (PMC10257615; doi:10.1007/s00431-023-04915-3)
Supplement: Supplementary file 1 — Supplementary file1 (DOCX 19 KB) [file 431_2023_4915_MOESM1_ESM.docx]

**Supplementary Table 1**: Detailed distribution of the fractured bones in the three different age groups.

|  | **0-5 years** | |  | **6-12 years** | |  | **13-17 years** | |
| --- | --- | --- | --- | --- | --- | --- | --- | --- |
|  | **n** | **%** |  | **n** | **%** |  | **n** | **%** |
| scaphoid | 0 | 0 |  | 7 | 1.8 |  | 17 | 5.9 |
| triquetrum | 0 | 0 |  | 0 | 0 |  | 1 | 0.3 |
| pisiforme | 0 | 0 |  | 1 | 0.2 |  | 1 | 0.3 |
| hamatum | 0 | 0 |  | 0 | 0 |  | 1 | 0.3 |
| metacarpal 1 | 0 | 0 |  | 17 | 4.2 |  | 21 | 7.2 |
| metacarpal 2 | 1 | 1.6 |  | 6 | 1.5 |  | 5 | 1.7 |
| metacarpal 3 | 3 | 4.7 |  | 5 | 1.2 |  | 7 | 2.4 |
| metacarpal 4 | 2 | 3.1 |  | 5 | 1.2 |  | 4 | 1.4 |
| metacarpal 5 | 0 | 0 |  | 28 | 6.9 |  | 30 | 10.3 |
| proximal phalanx 1 | 7 | 10.9 |  | 46 | 11.3 |  | 31 | 10.7 |
| proximal phalanx 2 | 1 | 1.6 |  | 9 | 2.2 |  | 5 | 1.7 |
| proximal phalanx 3 | 5 | 7.8 |  | 13 | 3.2 |  | 11 | 3.8 |
| proximal phalanx 4 | 1 | 1.6 |  | 20 | 4.9 |  | 7 | 2.4 |
| proximal phalanx 5 | 9 | 14.1 |  | 122 | 30.0 |  | 29 | 10.0 |
| middle phalanx 2 | 2 | 3.1 |  | 13 | 3.2 |  | 12 | 4.1 |
| middle phalanx 3 | 1 | 1.6 |  | 16 | 3.9 |  | 18 | 6.2 |
| middle phalanx 4 | 2 | 3.1 |  | 19 | 4.7 |  | 24 | 8.3 |
| middle phalanx 5 | 3 | 4.7 |  | 36 | 8.8 |  | 28 | 9.7 |
| distal phalanx 1 | 8 | 12.5 |  | 11 | 2,7 |  | 3 | 1.0 |
| distal phalanx 2 | 6 | 9.4 |  | 5 | 1.2 |  | 2 | 0.7 |
| distal phalanx 3 | 6 | 9.4 |  | 15 | 3.7 |  | 18 | 6.2 |
| distal phalanx 4 | 6 | 9.4 |  | 9 | 2.2 |  | 6 | 2.1 |
| distal phalanx 5 | 1 | 1.6 |  | 4 | 1.0 |  | 9 | 3.1 |
| **total** | **64** | **100** |  | **407** | **100** |  | **290** | **100** |
