# Supplementary material for: Distribution and pattern of hand fractures in children and adolescents
Source: Eur J Pediatr. 2023 Apr 5;182(6):2785–92. doi: 10.1007/s00431-023-04915-3 (PMC10257615; doi:10.1007/s00431-023-04915-3)
Supplement: Supplementary file 2 — Supplementary file2 (DOCX 14 KB) [file 431_2023_4915_MOESM2_ESM.docx]

**Supplementary Table 2**: Indications for surgical treatment

|  | **n** | **%** |
| --- | --- | --- |
| displacement | 89 | 73 |
| open fracture | 10 | 8.2 |
| malrotation | 6 | 4.9 |
| intraarticular fracture | 6 | 4.9 |
| secondary displacement | 4 | 3.3 |
| (subtotal) amputation | 3 | 2.5 |
| pseudoarthrosis | 2 | 1.6 |
| dislocation | 1 | 0.8 |
| not specified | 1 | 0.8 |
| **total** | **122** | **100** |
